
JOURNAL INFORMATION
==============================
NLM Title Abbreviation: Inter Econ
Journal ID: Inter Econ
NLM Journal ID: 101086399

Inter Economics

ISSN: 0020-5346

ARTICLE INFORMATION
==============================
PMCID: PMC7584858
DOI: 10.1007/s10272-020-0918-9
Article ID: 918
Article version: 1
Subjects: Forum

Eurozone Output Gaps and the COVID-19 Shock

Brooks Robin rbrooks@iif.com

Fortun Jonathan jfortun@iif.com

Institute of International Finance, 1333 H St NW, Suite 800E, 20005-4770 Washington, DC, USA
Electronic publication date: 2020 Oct 24
Print publication date: 2020
Volume: 55
Issue: 5
First page: 291
Last page: 296
Copyright: © The Author(s) 2020
License: Open Access: This article is distributed under the terms of the Creative Commons Attribution 4.0 International License (https://creativecommons.org/licenses/by/4.0/).
Open Access funding provided by ZBW — Leibniz Information Centre for Economics.
License URL: https://creativecommons.org/licenses/by/4.0/

issue-copyright-statement: © The Author(s) 2020

==============================
The authors wrote this article in a personal capacity. The views and opinions expressed herein are their own and do not necessarily reflect the official policy or position of the Institute of International Finance or any other organisation, employer or company. Assumptions made in the analysis are not reflective of the position of any entity other than the authors.

The eurozone was marked by economic divergence in the decade before the COVID-19 shock, something that was a starting point for our Campaign against Nonsense Output Gaps (CANOO), which we unveiled in Brooks and Fortun (2019a) over a year ago. In the years leading up to the pandemic, countries in the euro core had recovered quickly from the global financial crisis in 2008/9, while the periphery suffered stagnant and intermittent growth, leaving GDP in some cases well below pre-crisis levels. One reason for this divergence can be found in financial conditions, which tightened substantially for countries like Italy and Spain during the sovereign debt crisis in 2011/12. That tightening in financial conditions made a uniform recovery across the eurozone difficult and some echoes of this can be found in financial markets today. Concerted ECB action has prevented real yields on the euro periphery from rising materially, but it is still the case that real Italian bond yields exceed those in the euro core. As a result, the potential for continued economic divergence within the eurozone exists, which may also exacerbate deflation risk for the region overall.

Figure 1

**Real GDP levels**

index, Q1 2008 = 100

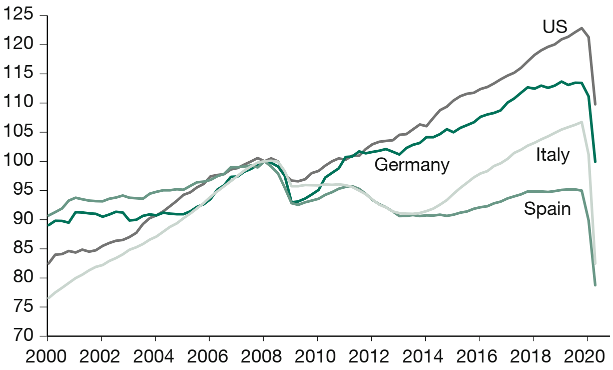

Note: Last data point is Q2 2020.

Sources: Haver Analytics and Bloomberg.

Figure 2

**Real interest rates**

10-year real yields, in %

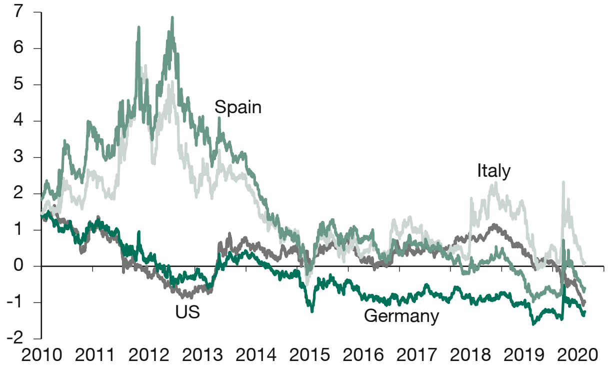

Note: Nominal yields minus inflation breakevens.

Sources: Haver Analytics and Bloomberg.

Figure 3

**Real GDP and potential GDP estimates for Spain**

index, Q1 2008 = 100

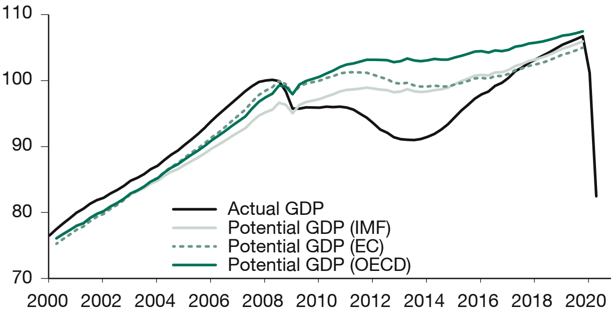

Note: Estimates for potential GDP from 2019.

Source: Haver Analytics.

Figure 4

**Real GDP and potential GDP estimates for Italy**

index, Q1 2008 = 100

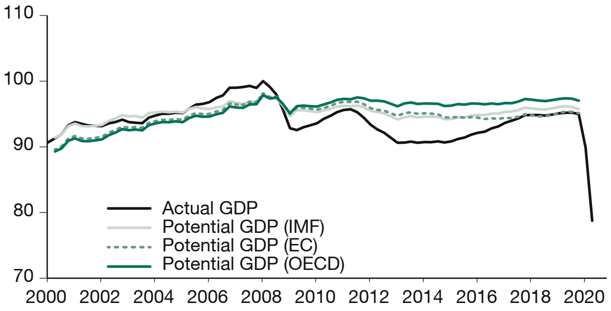

Note: Estimates for potential GDP from 2019.

Source: Haver Analytics.

Figure 5

**Real versus potential GDP growth**

in %

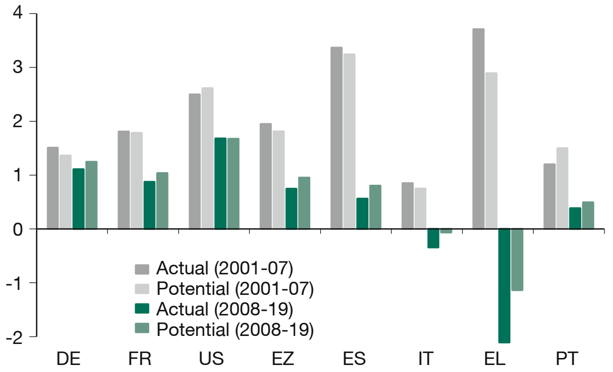

Note: Average annual GDP growth in 2001-07 and 2008-19 versus "consensus" estimates of potential GDP growth (average of IMF, EC & OECD).

Source: Haver Analytics.

Figure 6

**Production function approach**

in percentage points

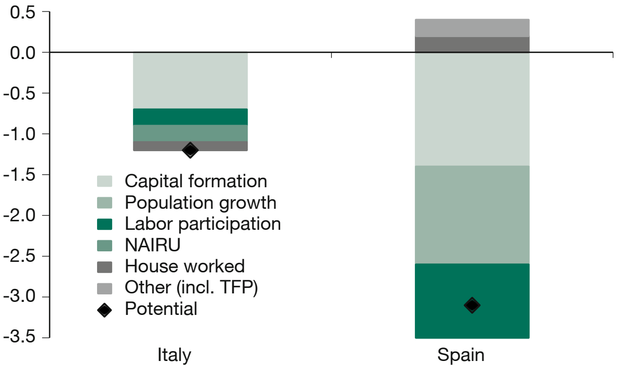

Note: Contributions to lower potential GDP growth (black) in the EC production function approach: 2010-18 vs 2001-7.

Source: Haver Analytics.

### Cyclical weakness on the euro periphery before COVID-19

The pace of eurozone recovery was highly uneven after the global financial crisis in 2008/9. Countries in the euro core recovered quickly, including Germany, while the periphery suffered prolonged stagnation and in some instances – Italy being the most notable example – still had GDP below 2008 levels in the run-up to the COVID-19 shock (Figure 1). One driver of this divergence is found in financial conditions. We calculate real ten-year sovereign bond yields for the US, Germany, Italy and Spain using inflation breakevens to deflate nominal bond yields (Figure 2). This exercise shows that the real yield for Italy and Spain rose to punitive levels in 2011/12, even as the real yield for Germany fell to what at the time were unprecedentedly low levels. This divergence in financial conditions made a uniform recovery across the eurozone difficult and is an important explanation for why Italy and Spain suffered a decade of stagnation after the global financial crisis. The reason this explanation is so important is because there is a lot of disagreement about whether it is structural or cyclical factors that are behind the depressed activity in periphery countries. The fact that financial conditions tightened so much puts us firmly in the cyclical camp, i.e. we believe that the slump on the periphery reflects depressed demand rather than structural constraints.

This view is at odds with 'consensus' estimates for potential GDP from before COVID-19, which 'bend down' in the case of Spain (Figure 3) and Italy (Figure 4). Our discussion here is not a criticism of these potential GDP estimates. Rather, it should be understood as pointing to how difficult it is to disentangle structural from cyclical factors when demand is depressed for long periods of time. To illustrate this, we calculate actual versus potential GDP growth rates for two windows: 2001 to 2007, i.e. before the global financial crisis, and 2008 to 2019, i.e. the aftermath of the Great Recession. Figure 5 shows average annual growth rates during these two windows, where we average potential growth estimates from the OECD, the IMF and the European Commission (EC) as an admittedly imperfect proxy for consensus. This shows that potential growth estimates closely track actual GDP growth, i.e. may not adequately capture 'potential' or what might have been possible under alternative policy settings. More specifically, these estimates of potential may not reflect growth that could have materialised had financial conditions for Italy and the rest of the periphery not tightened so much in 2011/12. In short, consensus estimates of potential are really much closer to realised 'trend' growth in different time windows, as opposed to potential.

The fact that estimates of potential track actual GDP so closely is not simply due to the widespread use of statistical techniques like the Hodrick–Prescott filter, which are essentially moving averages of actual GDP. The reasons run deeper than that. Figure 6 uses our approximation of the European Commission production function approach to decompose the slowdown in estimated potential growth in our two time windows into the underlying drivers for Italy and Spain. In Italy, potential growth is estimated to have fallen by slightly more than a full percentage point from the 2001 to 2007 window to the 2008 to 2019 window. The principal driver of that slowdown is capital formation, i.e. investment, followed by labour force participation, which declines. In Spain, potential growth is estimated to have slowed by a much larger amount, close to three percentage points. Again, the main driver by far is capital formation, with population growth and labour force participation as the other important drivers. All these things, especially investment, can be thought of as endogenous to financial conditions, i.e. the real interest rate. Given that the real interest rate was allowed to rise to punitive levels in Italy and Spain during the 2011/12 sovereign debt crisis, the fall in capital formation should most certainly not be seen as a 'structural' phenomenon, but instead as a largely cyclical response to tighter financial conditions and depressed demand.

### Low core inflation on the euro periphery

The ultimate arbiter in all this is inflation. If there was indeed a cyclical element to depressed GDP levels prior to COVID-19, it should be the case that estimates of underlying inflation – cleaned for temporary shocks – are low. Figure 7 shows a core harmonised index of consumer prices (HICP) inflation across the eurozone, where we use the year-over-year rate of annual average inflation to highlight slow-moving trends and filter out short-lived distortions. The grey area tracks the minimum and maximum of core inflation across countries in the eurozone. Core inflation in Italy and Spain was towards the upper end of the eurozone range before the global financial crisis; it spent the years since the sovereign debt crisis, however, at the lower end of that range. Meanwhile, core inflation in Germany was trending steadily higher in the run-up to the COVID-19 shock, even if that run-up was slow in the global disinflationary environment. Figure 8 takes a detailed look at the cross-section of core inflation in the eurozone. It shows the Q2 2020 readings of year-over-year annual average core HICP inflation, i.e. the end-points of the series shown in Figure 7. Countries for which financial conditions did not tighten in 2011/12, including the Netherlands, Austria, Belgium, Germany and France, were recording comparatively high inflation in the run-up to COVID-19. Meanwhile, Spain, Italy, Portugal and Greece saw low inflation rates,

Figure 7

**Core HICP inflation in the eurozone, 2000-20**

year-over-year rate, in %

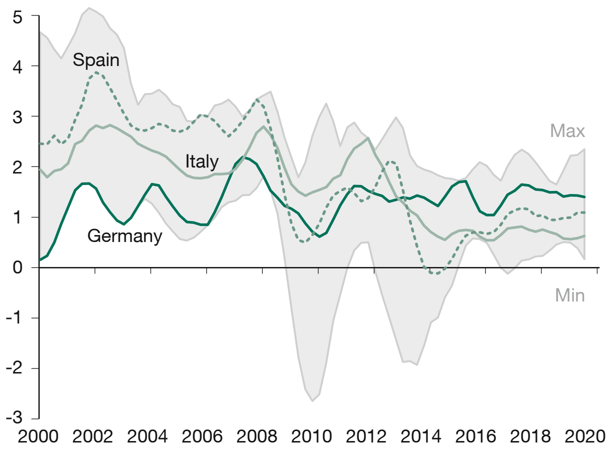

Note: Annual average. Last data point is Q2 2020.

Source: Haver Analytics.

Figure 8

**Core HICP inflation in Q2 2020**

year-over-year rate, in %

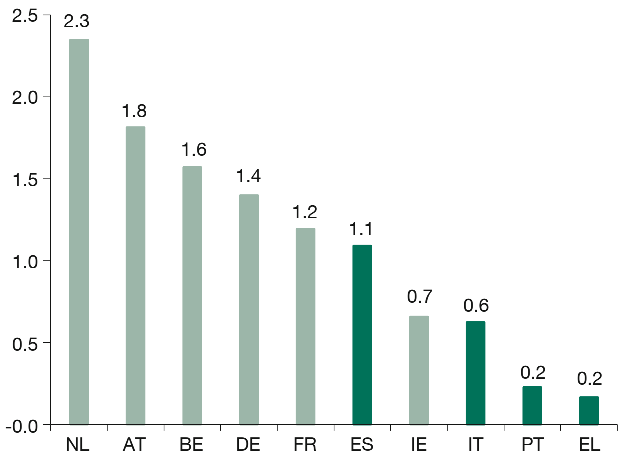

Note: Annual average.

Source: Haver Analytics.

consistent with the idea that low GDP levels in the run-up to the virus had a heavy cyclical component, i.e. were due to depressed demand.

In follow-up research to our output gap work, we use Phillips curves to compare consensus estimates of economic slack with trends in core inflation, starting with Brooks and Fortun (2019b). The idea here is simple. If estimates of diminishing slack are correct, they should coincide with rising inflation, once we control for other shocks like currency and commodity price moves. If core inflation remains too subdued relative to estimates of diminishing slack, this could be a sign that consensus unemployment or output gaps may be too conservative and that more slack exists. Figure 9 shows what this looks like for Spain, where the horizontal axis shows year-over-year

Figure 9

**Spain: Phillips curve since 1999 using European Commission estimates for the unemployment gap**

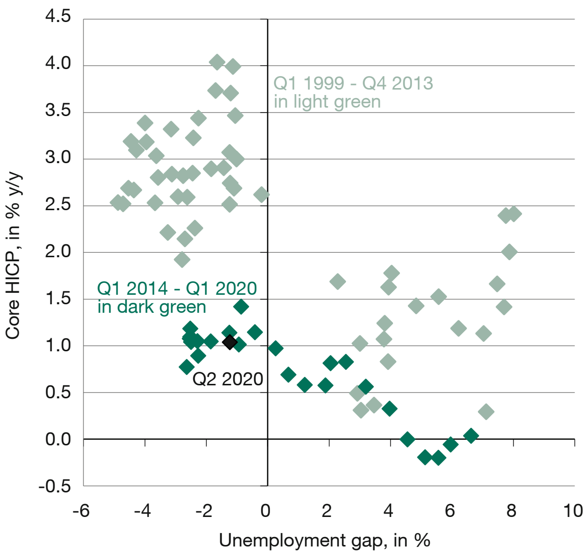

Source: Haver Analytics.

Figure 10

**Italy: Phillips curve since 1999 using European Commission estimates for the unemployment gap**

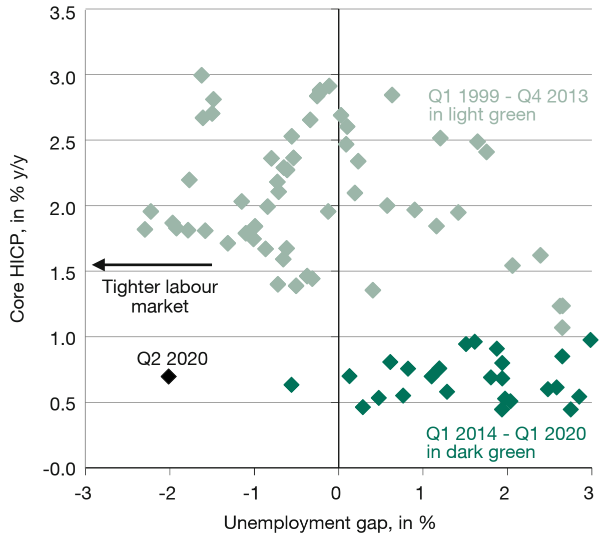

Source: Haver Analytics.

Figure 11
**Real interest rates**
in %

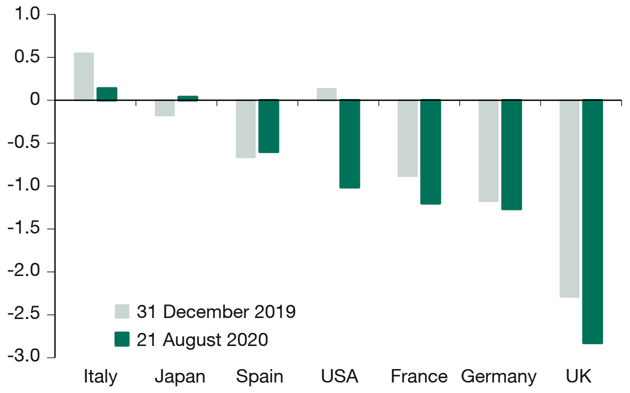

Note: Real 10-year sovereign bond yields (nominal - inflation breakevens).

Source: Haver Analytics.

Figure 12
**ECB inflation forecasts**
year-over-year rate, in %

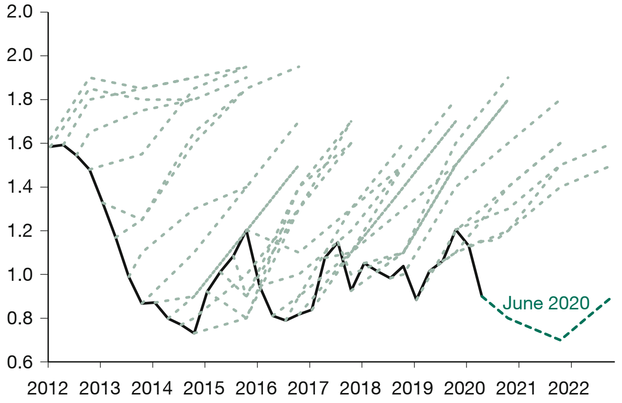

Note: ECB forecast for core inflation, March 2012 to June 2020.

Source: Haver Analytics.

core HICP inflation, based on quarterly average data, and the horizontal axis shows the unemployment gap, which is based on non-accelerating inflation rate of unemployment (NAIRU) estimates from the European Commission. The scatter plot highlights that core inflation remains very low relative to a labour market that at this point is considered 'tight', suggesting that more slack may remain or that NAIRU is potentially a lot lower. A similar story holds for Italy, as Figure 10 shows. Indeed, the COVID-19 shock has exacerbated the decoupling of core inflation and the consensus unemployment gap because declines in labour force participation have resulted in a 'spurious' decline in the unemployment rate. The Phillips curve framework suggests that the unemployment gap, based on still depressed core inflation, is still sizable and that NAIRU is potentially far below where consensus estimates put it. For the eurozone in aggregate, we have estimated that NAIRU may well be three full percentage points below the consensus estimate, which we believe lies around 8%, as flagged in Brooks and Fortun (2019c). Doing this same exercise for output gaps, Brooks, Heimberger and Tooze (2020) show that towards the end of 2019, Italy still had an output gap of 7%, while Spain had an output gap of 5%. In other words, key economies on the euro periphery went into the COVID-19 shock with large, pre-existing output gaps, which have since grown due to the severity of the economic shock.

## Conclusion

The reason this matters at the current juncture is because financial markets contain echoes of 2011/12. While concerted ECB action has prevented the kind of rise in real yields that took place during the sovereign debt crisis, it is the case that real yields in Italy and Spain are higher than in the eurozone core, including in France and Germany (Figure 11). As a result, it is possible that the COVID-19 shock is sowing the seeds for continued economic divergence within the eurozone and – as a result – mounting deflation risk for the single currency area overall. For European policymakers, this issue is one of first-order importance and one they have embraced. Key policymaking institutions are revisiting their output gap estimates, as Brooks and Fortun (2019d) document, and the European Commission has announced that its budget rules will remain suspended through the end of 2021, a welcome development given how elevated uncertainty is likely to remain for the foreseeable future. There are also policy implications beyond fiscal budgeting. Eurozone core inflation has failed to rise for many years and part of the underlying issue may be that economic slack was greater than consensus estimates allowed. Figure 12 shows the evolution of core HICP inflation and ECB core inflation forecasts over the years. If the COVID-19 shock ends up exacerbating the degree of economic divergence within the eurozone, this could add to the low inflation dynamic in the single currency area, increasing the need for additional monetary policy accommodation. The very latest ECB forecast certainly shows core inflation stuck at a very low level.


Robin Brooks, Institute of International Finance, Washington, DC, USA.

Jonathan Fortun, Institute of International Finance, Washington, DC, USA.


References

Brooks, R. and J. Fortun (2019a, 23 May), Campaign against Nonsense Output Gaps (CANOO), Institute of International Finance, Global Macro Views, https://bit.ly/3j8dneE (4 September 2020).
Brooks, R. and J. Fortun (2019b, 8 November), Inflation-consistent NAIRUs for Italy and Spain, Institute of International Finance, Global Macro Views, https://bit.ly/2FQJYHq (4 September 2020).
Brooks, R. and J. Fortun (2019c, 31 October), Reverse-Engineering the Euro Zone NAIRU, Institute of International Finance, Global Macro Views, https://bit.ly/32gy2pX (4 September 2020).
Brooks, R. and J. Fortun (2019d, 3 October), The Growing Debate over Output Gaps, Institute of International Finance, Global Macro Views, https://bit.ly/3j8dzdS (4 September 2020).
Brooks, R., P. Heimberger and A. Tooze (2020), Inflation-Consistent Output Gaps, Mimeo.
